# Domingo Marcolino Braile (1938-2020) Surgeon - Scientist - Professor - Businessman - Aviator The last flight of a great man

**DOI:** 10.21470/1678-9741-1-2020-0605

**Published:** 2020

**Authors:** Enio Buffolo, José Wanderley Neto, José Teles de Mendonça, Ricardo C. Lima, Paulo Roberto Brofman, Fernando A Lucchese, Fabio B Jatene

**Affiliations:** 1 Prof. Titular da Discipline of Cardiovascular Surgery, Hospital São Paulo, Escola Paulista de Medicina, Universidade Federal de São Paulo - EPM-UNIFESP, São Paulo, SP, Brazil.; 2 Ex-Presidente da Sociedade Brasileira de Cirurgia Cardiovascular Brasileira (SBCCV), São Paulo, SP, Brazil.; 3 Diretor do Instituto de Doenças do Coração de Alagoas (IDC), Maceió, AL, Brazil.; 4 Cardiovascular Surgery Division, Universidade Federal de Sergipe (UFS), Aracaju, SE, Brazil.; 5 Division of Cardiovascular Surgery of Pronto-Socorro Cardiológico de Pernambuco - PROCAPE, Recife, PE, Brazil.; 6 Cardiovascular Surgery Division, Pontifícia Universidade Católica do Paraná (PUCPR). Curitiba, PR, Brazil.; 7 Cardiovascular Medicine and Surgery; Faculdade Federal de Ciências Medicas, Porto Alegre, RS, Brazil.; 8 Cardiovascular Surgery Division, Instituto do Coração do Hospital das Clínicas da Faculdade de Medicina da Universidade de São Paulo (InCor-HC-FMUSP), São Paulo, SP, Brazil.

Brazil lost a great Brazilian. In a moment of strong worldwide commotion, our beloved Domingo Marcolino Braile left us. A few months before, his 500-page biography, A céu aberto, was published, just a little register of a life full of achievements and service to the Brazilian cardiology and to the country.

Brave, humble, passionate about medicine and the human being, Dr. Braile epitomizes the greatness of cardiovascular surgery. Being a part of the first generation of cardiovascular surgeons, he could establish himself wherever he wanted or seemed comfortable to carry out his mission. However, he preferred to return to São José do Rio Preto and set up one of the most creative and innovative cardiology centers in the world. This achievement facilitated the internalization of cardiac surgery in Brazil, directly assisting in the installation of 21 cardiac surgery services in the country.

Studious and dedicated, Dr. Braile became a reference and made his knowledge accessible to everyone. His generosity translates into the improvement of the quality of care of countless groups of cardiovascular surgeons who have set up in Brazil. He took care to provide the country with basic inputs for safety and quality in surgeries. Cardiopulmonary bypass machines, oxygenators, prosthetic heart valves, grafts, supplies for myocardial protection, aortic stents and catheter valves, the latter two of neat and advanced technology competing with more developed countries and allowing Brazilian cardiology to be at the side and sometimes ahead, without gaps, of global cardiovascular surgery. By developing national technology, he made cardiac surgery possible in the country without the high cost of imported equipments.

Until his farewell, even at home, in an adapted ward, Dr. Braile continued fighting for his life working on challenges and projects in the cardiovascular area.

As a cardiovascular surgeon, he accepted the challenge of installing a state-of-the-art service outside the national capitals, making São José do Rio Preto, through the Institute of Cardiovascular Diseases (IMC), a reference in medical assistance and quality.

The period as a cardiovascular surgeon was fruitful: Dr. Braile published 450 scientific articles and wrote 25 book chapters.

The associative work has been remarkable since the creation of the Cardiac Surgery Department of the Brazilian Society of Cardiology (SBC), later Brazilian Society of Cardiovascular Surgery (SBCCV), of which he was president. Dr. Braile made Teaching his most expressive mark: he was a true master. At the academy, he received his PhD degree in 1990 at Federal University of São Paulo (Unifesp) and was Professor at the Faculty of Medicine of Catanduva and at the State University of Campinas (Unicamp). He was also one of the founders of the Faculty of Medicine of São José do Rio Preto (Famerp).

The love for his city and the community made him a character beyond medicine.

By teaching he became the main responsible for the development and equalization of Brazilian cardiovascular surgery. With him, we learn that: “TO LEARN IS EASY, as long as we have someone willing to teach us”. Always ready to collaborate with colleagues, he had a great ability to value the youngest, for whom he always had a word of encouragement, guidance and support.

Later, in a more mature phase, when he had done everything in the operative field and acquired enviable experience and knowledge in the field of cardiac surgery, Braile started a new project, dedicating all his intellect, wisdom and hours of work in the development of our Brazilian Journal of Cardiovascular Surgery (BJCVS). It took years of full dedication, leading BJCVS to achieve the highest degree of quality and scientific recognition. He was tireless in this project, working with admirable devotion only seen in special men like Braile.

Braile was honored as an honorary citizen of several Brazilian states for the relevant services provided to Brazilian cardiology. His academic curriculum is encyclopedic. His contribution is massive.

We cannot fail to register his extraordinary love for aviation. He piloted gliders and multi-engine aircrafts alone or with his family, under Instrument Flight Rules since 1955. Since its introduction, he has used GPS, a device that revolutionized modern aviation.

Braile has become a mark of work, honesty, friendship, complicity, solidarity and love to others.

We were all privileged to live with him, to be his disciples and to become his friends; as he liked to emphasize, friend-brothers.

We had a group of close “brothers” who traveled every year and it was agreed that, far from the borders of Brazil and the strenuous routine of cardiac surgery, it was the opportunity to think about cardiovascular surgery and our country, and how we could improve results and better train the crowd of young enthusiasts of Brazilian cardiovascular surgery. Disagreements were common in these conversations, ideas were formulated; everything ended up converging on the uncompromising defense of Brazil and our cardiovascular surgery. The final brushstroke in our works and dreams was always his. “Negão” (his nickname) was our guide and our safe haven. Just as he instilled ideas in our minds, he accepted others, embracing them as if they were his ideas. He was always open to learn, stimulate and encourage the youngest.

Magnanimous, wise without being arrogant, reasonable without being subservient, affectionate and paternal when it was necessary and severe when the occasion demanded it, Dr. Braile represented for us a brother, father, friend, teacher and student; above all, an example to be followed. This guy was the “Brazilian Cardiovascular Surgery Guy”.

Our last image of Dr. Braile was when we visited him during his illness. Immobilized on a bed in his house, he received us for almost a whole day. The countenance deconstructed by the disease soon recovered in a conversation that derived from the disease to the future, always imagining scenarios and projects that could improve people’s lives. One of them, which we have already started, was the creation of the Brazilian Academy of Cardiovascular Surgery, which seeks to preserve the memory of cardiac surgery and our pioneering heroes. He will be the patron of this institution.

Finally, among his characteristics, which lack adjectives to point out, we highlight the great work of the family constitution in the figure of Maria Cecília (his wife), Valéria and Patricia (his daughters), sons-in-law and the 4 grandchildren who formed the solid base of the Braile saga.

The poem “Consoada”, by Manuel Bandeira, fits well for Dr. Braile’s epic existence:

*“When the Unwanted Guest arrive*
*(I don’t know if harsh or gentle),*
*maybe I’m afraid.*
*Maybe I smile, or say:*
*- Hello, inescapable!*
*I had a good day, the night may come down.*
*(The night with its spells.)*
*It will find the field plowed, the house clean,*
*The table set,*
*With everything in its place.”*
*So, it was Braile.*

## Figures and Tables

**Fig. 1:**
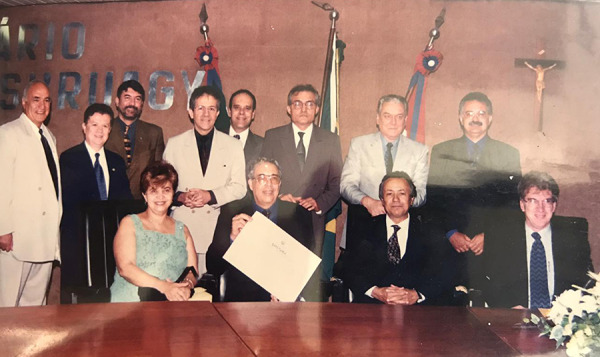
Domingo Braile receiving the title of honorary citizen of Alagoas in the company of colleagues and friends.

**Fig. 2:**
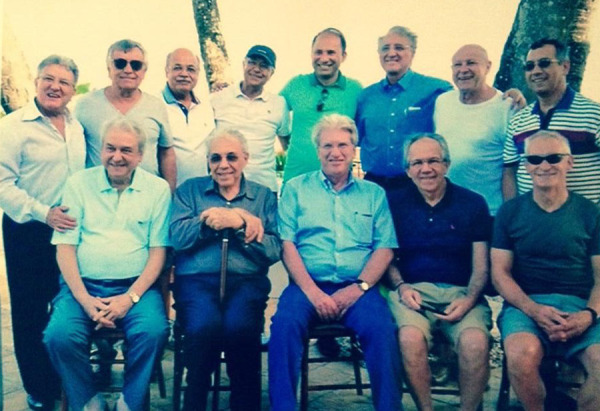
Relaxed with a group of colleagues gathered at Wanderley’s house in Maceió.

